# The scientific standing of nations and its relationship with economic competitiveness

**DOI:** 10.1371/journal.pone.0304299

**Published:** 2024-06-20

**Authors:** Giovanni Abramo, Ciriaco Andrea D’Angelo

**Affiliations:** 1 School of Technological Sciences and Innovation, Universitas Mercatorum, Rome, Italy; 2 Department of Engineering and Management, University of Rome “Tor Vergata”, Rome, Italy; Max Planck Institute for Solid State Research, GERMANY

## Abstract

In the current knowledge-based economy, the abilities of the national research system are a key driver of the country’s competitiveness and socio-economic development. This paper compares the scientific standing of the OECD countries and eight other relevant economies. We use a bibliometric indicator of research performance, applied first at the individual level. This approach avoids the distortions of the aggregate-level analyses extant in literature and practice, which overlook the different publication intensities across research fields. We find a strong correlation between research performance and the economic competitiveness of nations and a moderate but significant correlation between research performance and the propensity to spend on research.

## 1. Introduction

The importance of research as a driver of innovation, competitiveness, and socio-economic development has been amply demonstrated, beginning with Robert Solow’s 1956 milestone work [1] and the countless subsequent studies [2–7]. On this basis, with ever more conviction, governments across OECD countries and partner economies have applied financial and non-financial instruments to stimulate investment in research and increase the effectiveness and efficiency of national research systems.

Over several years, economists and bibliometricians have developed and applied indicators and models that can effectively portray a nation’s research profile. Indicator systems, some of which do not rely solely on publication data, were formulated by entities such as the U.S. National Science Foundation and the UK government as early as the 1950s (refer to [8] for a historical examination of the development of science and research taxonomies, along with statistical insights). Additionally, various nations initiated assessments of their scientific and technological competitiveness during the 1970s. Subsequently, routine reporting systems were established; for instance, the Office of Science and Technology in France and the Ministry of Research and Technology in Germany implemented such systems in the late 1980s and 1990s. Nations’ research performance rankings have been produced to inform decisions at different levels. Research-based multinational corporations that can access reliable accounts of the different countries’ scientific standing can better inform their strategies for locating R&D activities. For the governments, such reports enable checks on the effectiveness of policies and initiatives in support of research, adjustments in light of the strengths and weaknesses of the different scientific fields, and effective allocation of resources. The publication of performance rankings can also stimulate continuous improvement within institutions and at the levels of individual research teams and researchers.

The resort to citation-based indicators to measure the scientific standing of nations began with the pioneering work by Robert May in 1997 [9], after which several scholars followed suit [10–15]. These scholars have gradually expanded the number of countries and research fields analyzed, and refined bibliometric performance indicators. Some scholars have attempted to free the performance scores from their size dependency by dividing the overall bibliometric score by some measure of input (total number of researchers, R&D expenditures, or GDP), ignoring though that publication intensity varies across research fields [16].

This study aims to overcome some limitations of the methods and indicators adopted so far by starting with performance measurement for individual researchers. This involves identifying the researchers in each country (disambiguating their real identity), their publications in a period, their contributions to publications, and their prevailing field of research; next, measuring the total scholarly impact of each and comparing it with that of all other researchers in the world in the same field and period. The performance of a country in a field or area will then be given by the average of the normalized performance of the country’s researchers in that field or area and the overall performance by the average of the normalized performance of all researchers in the country.

In this paper, we present the proposed method and relevant scientific performance indicator, applying them for the calculation of performance scores and rankings in the pre-Covid 2015–2019 period for the 38 OECD countries and eight non-member economies (chosen by economic or scientific significance: China, India, Brazil, Russia, Taiwan, Argentina, Singapore, and South Africa), in 222 research fields, 11 research areas and overall. The authors already applied the same indicator to compare the research performance of the USA and Russia [17]. Computation problems represent the challenge here due to the scale of the analysis. The rankings of nations provided in each research field, area, and overall should convey valuable information for the above-mentioned stakeholders of research systems.

Furthermore, in this work, we investigate the possible association between a country’s scientific standing and i) its propensity to invest in R&D, as measured by gross domestic expenditure on R&D (GERD) as a percentage of GDP [18]; and ii) its economic competitiveness as measured by labour productivity [19].

## 2. The literature on evaluating a nation’s scientific standing

Assessing a country’s scientific standing at a field level presents a formidable challenge [20,21]. Definitions of “scientific standing” vary, and there is no consensus on the right approach to gauge it. Nevertheless, scientific standing inherently involves making comparisons and achieving superiority in terms of quality [22].

The fundamental question is understanding the concept of research quality and whether it differs from research impact. Some argue that impact is merely one dimension of research quality, with other dimensions including relevance and research rigor [23–25]. Conversely, some claim that quality and impact are separate components of scientific standing [26].

A chronological analysis of studies applying citation-based metrics to evaluate a nation’s scientific standing reflects the evolution of bibliometric indicators and methodologies in recent years. May’s landmark study in 1997 compared the scientific standing of 15 nations in STEM fields over a 14-year period using indicators such as WoS-indexed publications, citations, and citations per unit of spending. Subsequently, Adams [10] compared England’s performance in 47 fields with that of six other countries. An extended analysis by King [11] involved 31 countries over a decade, introduced additional bibliometric indicators, and used field-normalized citations to account for country size differences. Cimini et al. [14] used citations to scientific articles to assess the scientific standing of 238 countries in 27 scientific domains and 307 subdomains. More recently, Patelli et al. [27] applied a framework leveraging on the Economic Fitness and Complexity algorithm [28] to quantify the scientific standing of nations and regions.

Alongside, research on assessing a nation’s relative research standing has increasingly focused on excellence, particularly as measured by highly cited articles (HCAs). HCAs offer a transparent framework for domestic and international comparisons [29]. Bornmann and Leydesdorff [12] developed a mapping approach to locate field-specific centers of excellence worldwide, while Pislyakov and Shukshina [30] utilized HCAs to identify “centers of excellence” in Russia. Abramo and D’Angelo [15] introduced an innovative output-to-input methodology enabling the assessment of research strengths and weaknesses by considering the ratios of leading scientists and HCAs to research funding in specific fields. Furthermore, publications like the Nature Index Annual Tables and organizations like the CWTS of the University of Leiden and the SCImago group have provided for long time yearly country rankings based on total publications, mean field-normalized citations per article, and the share of HCAs.

## 3. Methods and data

A significant concern regarding the approaches found in the literature is the need for more adoption of efficiency indicators, which account for output relative to input. Many studies either do not incorporate efficiency indicators or do so at the aggregate level, i.e. dividing total output or impact by total research expenditures or by total number of researchers, overlooking the different throughput across fields (for example, mathematicians will produce less than clinicians, and within the latter, vascular surgeons will produce less than haematologists). Consequently, the studies that do not account for input generally rank the USA at the top in most scientific fields. Still, they do not clarify whether the USA’s high ranking results from more significant research investment or superior scientific performance. Those that divide the overall output or impact by total R&D spending lead to unreliable performance scores.

To circumvent the problem of lack of input data, size-independent indicators like “average normalized citations per publication” or MNCS were introduced [31,32]. Unfortunately, they prove ineffective, violating a key principle of production theory: if output increases with constant input, performance should not decline. With the “average normalized citations per paper” approach, this violation occurs when organizations or individuals produce additional publications with even slightly lower normalized impact than their previous average [33].

To the best of our knowledge, as far as national-level rankings are concerned, besides Italy [34], successful attempts to account for inputs when evaluating individual, field, and institutional performance are limited to Norway [35] and Sweden [36]. The first two studies use an approach based on actual input and output levels, while the latter relies on changes in input and output levels.

The availability of input data at the field level represents the main obstacle to measuring and comparing nations’ research performance. Our methodology is built on the premise that the strength of one research field in a country can be determined by the superior performance of researchers in that field compared to others. Since scientific publication rates vary across fields, a direct comparison of performance at the aggregate level (total number of publications or citations) would favor those countries specialized in fields with higher publication rates, leading to distorted results [37].

To address this, we proceed in four steps. First, we identify the researchers in each country and classify them in research fields on the basis of the prevalent domain of their publications. Second, we measure the total impact of a country’s researchers. Third, we normalize the performance of each researcher by the average performance of all world researchers in the same field. Finally, we average the field-normalized performance of all researchers in a country by field, area, and overall level, leading to the relevant world rankings.

### 3.1 Dataset construction

The analyses were conducted first at the individual level and then at the aggregate level by research field (subject category, SC), research area, country, and concerned the 2015–2019 period; a more recent period would have compromised the accuracy of the measurement of the publications’ scholarly impact. In fact, the larger the time citation window, the closer the measurement approximates the overall impact [38]. To identify the countries’ researchers, we recurred to the rule-based scoring and clustering algorithm by Caron and Van Eck, or CvE [39]. We applied it to the in-house Web of Science (WoS) database of the Centre for Science and Technology Studies (CWTS) at Leiden University (updated to the 13th week of 2022).

In the author disambiguation process carried out by Caron and Van Eck in 2014, bibliometric data related to authors and their publications is used as input to identify clusters of publications likely authored by the same individual. The CvE method comprises three main stages: In the initial pre-processing phase, author name blocks are generated to reduce computational workload in subsequent phases.

During the rule-based scoring and oeuvre identification phase, potential author oeuvres are determined. For each author name block, the associated publication-author combinations (PACs) are identified. The score for a pair of PACs is computed using four sets of scoring rules that involve comparing author information, publication details, source data, and analyzing citation relationships.

The final score for a pair of PACs is the sum of scores obtained from these different scoring rules. These scores are assigned based on expert knowledge and fine-tuned by evaluating their accuracy using a test dataset. Experimental thresholds are also applied to decide whether two PACs belong to the same author oeuvre.

Candidate author oeuvres are identified separately for each author name block. Consequently, in the post-processing stage, candidate author oeuvres are merged if they share the same email address. This process results in the creation of final author oeuvres, which are referred to as “clusters.”

When the final author oeuvres have been obtained, meta-data is generated for each of the associated clusters. Table 1 reports, for example, the information that the algorithm associates with the cluster of publications by Nees Jan van Eck.

**Table 1:** Description of the output information of the CvE algorithm for Nees Jan van Eck.

Field	Value
cluster_id	31863740
n_pubs	89
first_year	2005
last_year	2021
full_name	van Eck, NJ
last_name	Van Eck
first_name	Nees Jan
email	ecknjpvan@cwts.leidenuniv.nl
organization	leiden univ
city	leiden
country	netherlands

We use algorithmic output to identify each country’s research staff (i.e., by “country” field). Of course, this algorithm is not error-free, for example, in dealing with authors of very common names or those with highly diversified and heterogeneous bibliographies whose portfolios could be split into two or more clusters. Specifically, the CvE algorithm values precision over recall: if insufficient evidence exists for assigning publications to the same cluster, the method will assign them to different clusters. Consequently, an author’s publications may be split over multiple clusters. The validation of the algorithm conducted by the authors and based on two datasets of Dutch authors resulted in an average precision of 95% and an average recall of 90%, with the errors increasing for more common author names. Moreover, Tekles and Bornmann [40] found that the CvE algorithm was the best-performing approach compared to other unsupervised disambiguation approaches. Finally, it should be noted that for this paper, when moving to the aggregate level, errors in author disambiguation tend to compensate, being independent of the authors’ country, and so should have negligible effects on results.

To exclude “occasional” and terminated researchers and improve the robustness of the analysis, we exclude those clusters that fail to meet one or more of the following requirements over the 1980–2022 period:

- include at least ten publications (excludes “occasional” authors, for whom clustering has lower confidence levels);
- include at least one publication published in 2020 or later (designed to exclude researchers no longer active);
- with a “research activity” (measured by the difference between the first and the last publication year) of a minimum of five years (designed to include only “established” authors).

To assign authors to research fields, we adopt the WoS field classification scheme, consisting of 254 subject categories (SCs) falling into 13 areas. We associate each publication with the WoS SC of the hosting journal. We then identify the “prevalent” SC of the publications in a cluster. The prevalent SC will be the author’s research field. There occur rare cases of clusters with more than one prevalent SC (representing around 7% of total researchers). In such cases, the prevalent SC is randomly assigned among those with the highest frequency.

To avoid distortions due to WoS limitations in the coverage of literature, we limit the dataset to all SCs of the sciences and several SCs of the social sciences [41–43]. The field of observation then includes 222 SCs grouped in 11 areas. In such fields, we count 2,250,698 clusters/authors affiliated with 206 different countries. We limit the analysis to the 38 OECD countries and another eight non-OECD economies selected for economic/scientific relevance: China, India, Brazil, Russia, Taiwan, Argentina, Singapore, and South Africa.

**Fig 1:**
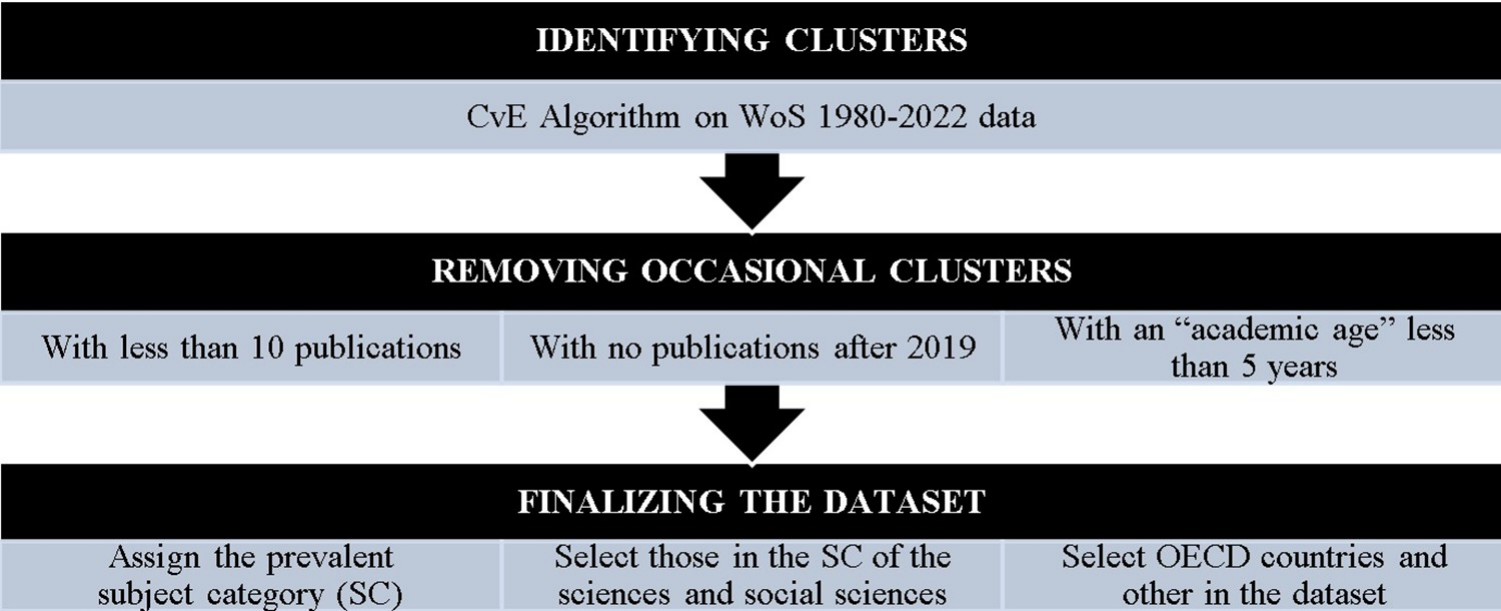
Workflow of the dataset construction.

Fig 1 illustrates the workflow for the data selection and refinement, while Table 2 shows the breakdown of clusters by area, for the main countries in the dataset and overall. The 46 countries considered total almost 2.1 million researchers, or almost 92.5% of the world’s total. Leading the way is the USA with just under half a million researchers, or 21.3% of the total, followed by China (242k, 10.7%), Japan and the UK (both with 110k researchers, or 4.9% of the total). Clinical Medicine is the area with the largest number of researchers worldwide, at just over 607k (or 27.0% of total), followed by Engineering (377k, 16.7%), Biology (346k, 15.4%), and Biomedical research (297k, 13.2%).

**Table 2:** Number of clusters (x 1000) by area, for seven main countries and overall.

Area	USA	China	Japan	UK	Germany	Italy	France	Tot. 38 OECD countries	Other 8 countries in the dataset	Total world
Biology	72.9	29.8	15.5	14.0	14.3	14.3	13.7	245.0	74.9	345.5
Biomedical Research	77.0	27.6	18.4	13.5	14.6	17.8	13.0	228.2	49.2	297.4
Chemistry	21.0	26.3	7.1	4.5	6.3	4.1	5.5	80.4	49.4	144.1
Clinical Medicine	161.1	25.0	37.2	37.6	29.9	34.0	22.1	506.0	66.9	607.4
Earth & Space Sciences	20.9	18.6	3.6	6.2	5.6	5.6	5.5	82.7	31.4	124.3
Economics	11.1	1.8	0.4	3.9	1.5	1.8	1.1	33.8	3.6	39.7
Engineering	48.0	79.8	14.4	12.8	15.2	13.1	13.7	210.8	125.9	376.7
Mathematics	8.0	6.5	1.3	1.8	2.1	2.3	2.6	30.1	11.0	46.0
Physics	33.7	24.9	11.7	9.2	14.0	9.2	10.3	132.8	54.9	203.5
Political & Social sciences	15.3	0.8	0.2	4.5	1.5	0.5	0.3	35.4	2.3	39.3
Psychology	11.4	0.6	0.3	2.1	1.6	0.9	0.6	24.9	1.4	26.8
Total	480.3	241.7	110.2	110.0	106.6	103.6	88.4	1610.1	471.0	2250.7

### 3.2 Measuring the scientific standing of a country

We measure the scientific performance of a country starting from the individual researcher through the Fractional Scientific Strength indicator or FSS_p_, defined as:

$${FSS}_{p}=\sum_{i=1}^{N}c_{i}\;f_{i}$$

where:

*N* = number of WoS publications by the author in the period under observation.

*c_i_* = impact of publication *i*. i.e. weighted average of the field-normalized citations received by publication *i* and the field-normalized impact factor of the hosting journal, as suggested in Abramo et al. [44] citations are normalized to the mean of the distribution referring to all cited publications of the same year and WoS SC of publication i. The impact factor of the journal refers to the year of publication and is normalized with respect to the average of the distribution of IFs of all journals in the same SC of publication *i*);

f_i_ = fractional contribution of the author to publication *i*, given by the reciprocal of the number of co-authors in the byline.

A thorough description of the methodology, assumptions and limitations, and underlying theory can be found in Abramo and D’Angelo [34].

Note that in the dataset there are 73,679 clusters with nihil FSS. These are:

- “inactive” researchers (with no eligible publications, i.e. articles, letters, reviews and conference proceedings), during the observation period (2015–2019),
- “active” researchers, but whose 2015–2019 eligible publications show nihil impact.

The performance of countries, that are heterogeneous in the research fields of their staff cannot be directly measured at the aggregate level [37]. So, after measuring the performance of individual authors (*FSS_p_*) we normalize individual performance by the average of the relevant SC world distribution. At the aggregate level then, the yearly performance *FSS*_*A*_ for the aggregate unit *A* (the national research staff in a SC/area/overall) is:

$${FSS}_{A}=\frac{1}{RS}\sum_{j=1}^{RS}\frac{{FSS}_{P_{j}}}{\bar{{FSS}_{P}}}$$

Where:

*RS* = number of authors in the unit, in the observed period;

${FSS}_{P_{j}}$ = performance of author *j* in the unit;

$\bar{{FSS}_{P}}$ = average performance of all world authors in the same SC of author *j*.

A value of *FSS_A_* = 1.20 means that the country unit A employs authors with average performance of 20% higher than expected, i.e. the world average.

In this way, we can measure the performance of a country at SC, area, and overall level, avoiding distortions due to the different intensities of publication and citation across SCs. For significance, the ranking lists include all and only the countries with at least:

- 30 clusters, for analysis at SC level,
- 100 clusters, for analysis at area level.

### 4. Results

Below, we will present the outcome of applying the proposed approach through some examples. For the research performance indicator and method adopted, one country will perform better than another if one or more of the following conditions occur: on average researchers are (i) better than others; (ii) devote more time to research; or (iii) have more resources available to them. The performance indicator used (Fractional scientific strength or FSS) is normalized to the world average; e.g. a score of FSS = 1.20 indicates a performance 20% higher than the world average.

A first application of the measurements made allows us to identify the scientific strengths and weaknesses of each country by field. As an example, in Table 3 we show the top ten and bottom ten research fields by research performance (FSS) of German researchers. Germany ranks top in three fields: Business; Soil Science; Mathematical & Computational Biology. At the same time, the country ranks bottom in four fields: Education, Scientific Disciplines; Materials Science, Composites; Engineering, Environmental; Materials Science, Characterization & Testing.

**Table 3:** Top ten and bottom ten research fields (SC) in Germany by research performance (FSS). For the sake of significance, the ranking lists contain all and only countries with at least 30 researchers in the field and research fields with at least five countries.

Research field	Res. Staff	FSS	Rank	Percentile
Business	163	1.824	1 out of 31	100
Soil Science	253	1.654	1 out of 23	100
Mathematical & Computational Biology	127	1.564	1 out of 13	100
Forestry	184	1.751	2 out of 31	97
Physics, Fluids & Plasmas	505	1.392	2 out of 25	96
Physics, Mathematical	225	1.378	2 out of 24	96
Horticulture	61	1.778	2 out of 23	95
Biochemistry & Molecular Biology	3467	1.330	3 out of 42	95
Radiology, Nuclear Medicine & Medical Imaging	2399	1.326	3 out of 36	94
Anthropology	113	1.314	2 out of 18	94
…				
Engineering, Aerospace	122	0.669	13 out of 15	14
Computer Science, Hardware & Architecture	54	0.432	7 out of 8	14
Ornithology	30	0.876	8 out of 9	13
Gerontology	104	0.720	8 out of 9	13
Medical Informatics	103	0.505	9 out of 10	11
Thermodynamics	98	0.447	17 out of 18	6
Education, Scientific Disciplines	77	0.863	7 out of 7	0
Materials Science, Composites	68	0.600	13 out of 13	0
Engineering, Environmental	36	0.282	8 out of 8	0
Materials Science, Characterization & Testing	32	0.259	5 out of 5	0

Complete data for all countries can be seen in *S1 File*.

Scaling up the aggregation level, we can observe the performance of each country in each research area. In this regard, Table 4 shows the scientific standing of New Zealand by area. The country ranks top in Biomedical research and close to the top in Psychology, and Clinical Medicine. In contrast, performance in Mathematics, Economics, and Political and social sciences is below the median.

**Table 4:** Research performance of New Zealand, by research area. For the sake of significance, the ranking lists contain all and only countries with at least 100 researchers in the area.

Research area	Res. Staff	FSS	Rank	Percentile
Biomedical Research	504	1.689	1 out of 42	100
Psychology	170	1.288	4 out of 27	88
Clinical Medicine	2118	1.227	7 out of 45	86
Physics	244	1.142	9 out of 43	81
Engineering	818	1.086	11 out of 44	77
Chemistry	216	1.070	12 out of 42	73
Biology	2065	1.109	15 out of 45	68
Earth and Space Sciences	739	0.920	19 out of 43	57
Political and social sciences	320	0.826	19 out of 36	49
Economics	375	0.888	21 out of 36	43
Mathematics	109	0.717	30 out of 39	24

Full data can be seen in *S2 File*.

Adopting a complementary perspective, the analysis can provide rankings of countries by research field and area. Table 5 shows the case of “Meteorology & Atmospheric Sciences”, where 33 of the 46 countries considered employ at least 30 researchers. Leading the ranking is Switzerland, with an average performance of its 199 researchers that is 58 percent above the world average (FSS = 1.580), followed by Portugal (1.359), the USA (1.273), and the UK (1.221). Conversely, Argentina, Hungary, Mexico, and finally Russia appear in the trailing positions.

**Table 5:** Research staff and research performance of countries in “Meteorology & Atmospheric Sciences”. For the sake of significance, the ranking lists contain all and only countries with at least 30 researchers in the research field.

Rank	Country	Res. Staff	FSS	Rank	Country	Res. Staff	FSS
1	Switzerland	199	1.580	17	Sweden	156	0.781
2	Portugal	43	1.359	18	Spain	301	0.772
3	United States	3776	1.273	19	Greece	119	0.760
4	United Kingdom	934	1.221	20	Finland	171	0.759
5	Australia	282	1.197	21	Japan	564	0.718
6	Netherlands	166	1.124	22	France	647	0.693
7	China	1086	1.118	23	India	475	0.678
8	Norway	147	1.021	24	Czech Republic	66	0.676
9	South Africa	45	1.007	25	Denmark	47	0.643
10	Germany	982	0.931	26	Poland	66	0.621
11	Canada	335	0.922	27	Brazil	173	0.577
12	Israel	53	0.911	28	Taiwan	81	0.497
13	Austria	96	0.893	29	New Zealand	63	0.475
14	Italy	317	0.844	30	Argentina	73	0.408
15	Belgium	93	0.790	31	Hungary	46	0.388
16	South Korea	157	0.787	32	Mexico	47	0.323
				33	Russia	431	0.260

Finally, Table 6 reports the performance of all 46 countries considered, obtained by averaging the FSS of all researchers in each one. Leading the way is Singapore, a relatively small country with 9501 researchers, operating with at least 30 researchers in 79 fields out of 222: average research performance is about 60 percent higher than the world average. Australia is second, followed by Denmark, Netherlands, Switzerland, UK and USA. At the tail end, in addition to Argentina, are four countries of the former Soviet Union: Lithuania, Slovakia, Russia, and Latvia. China, the second largest country with its over 240,000 researchers, places twelfth, ahead of Sweden and Germany; France and Japan fall below the median.

**Table 6:** The dataset’s research staff and overall research performance of countries.

Rank	Country	Res. Staff	No. of SCs*	FSS	Rank	Country	Res. Staff	No. of SCs*	FSS
1	Singapore	9501	79	1.606	24	Iceland	912	4	0.861
2	Australia	51720	171	1.463	25	India	65134	134	0.817
3	Denmark	15829	99	1.319	26	France	88405	159	0.797
4	Netherlands	39905	146	1.290	27	Taiwan	19851	109	0.766
5	Switzerland	25412	121	1.262	28	Spain	78949	174	0.748
6	United Kingdom	110039	196	1.256	29	Slovenia	3842	40	0.732
7	United States	480309	214	1.209	30	Greece	14551	96	0.732
8	Canada	62093	176	1.172	31	Estonia	1892	14	0.730
9	Belgium	18863	117	1.168	32	Chile	7851	73	0.671
10	New Zealand	7678	83	1.135	33	Japan	110215	154	0.656
11	Luxembourg	737	4	1.120	34	Poland	36865	133	0.627
12	China	241691	180	1.098	35	Costa Rica	377	1	0.612
13	Sweden	24868	130	1.092	36	Czech Republic	15785	107	0.606
14	Germany	106584	180	1.061	37	Brazil	64344	149	0.591
15	Ireland	7919	66	1.046	38	Hungary	8374	71	0.571
16	South Korea	41665	132	1.036	39	Colombia	3737	42	0.537
17	South Africa	9341	77	1.014	40	Turkey	39368	127	0.527
18	Austria	15106	105	0.959	41	Mexico	18999	99	0.457
19	Finland	13456	93	0.957	42	Lithuania	3071	29	0.447
20	Norway	13210	101	0.942	43	Argentina	13021	85	0.444
21	Italy	103604	164	0.902	44	Slovakia	5267	55	0.419
22	Israel	14940	100	0.897	45	Russia	48139	110	0.399
23	Portugal	16431	112	0.864	46	Latvia	1277	9	0.344

* With at least 30 researchers.

## 5. Discussion and conclusions

While assessing a country’s scientific standing is crucial for governments, businesses, and funding agencies as they determine their scientific priorities and allocate resources, it is also interesting to judge the extent of association between scientific standing and i) propensity to invest in research and ii) economic competitiveness.

The relationship between the scientific standing of nations and their propensity to invest in research while complex and multifaceted, is often characterized by a positive feedback loop, where a nation’s commitment to research can enhance its scientific standing, and a solid scientific standing can, in turn, encourage more significant investment in research. Robust scientific ecosystems attract talent, encourage innovation, and provide a knowledge base for further research. It is important to note that the relationship between scientific standing and research investment is not linear, and there can be variations among countries. Some nations may prioritize research as a means to improve their scientific standing, while others may initially invest in building a strong scientific foundation to attract further research investment.

**Fig 2:**
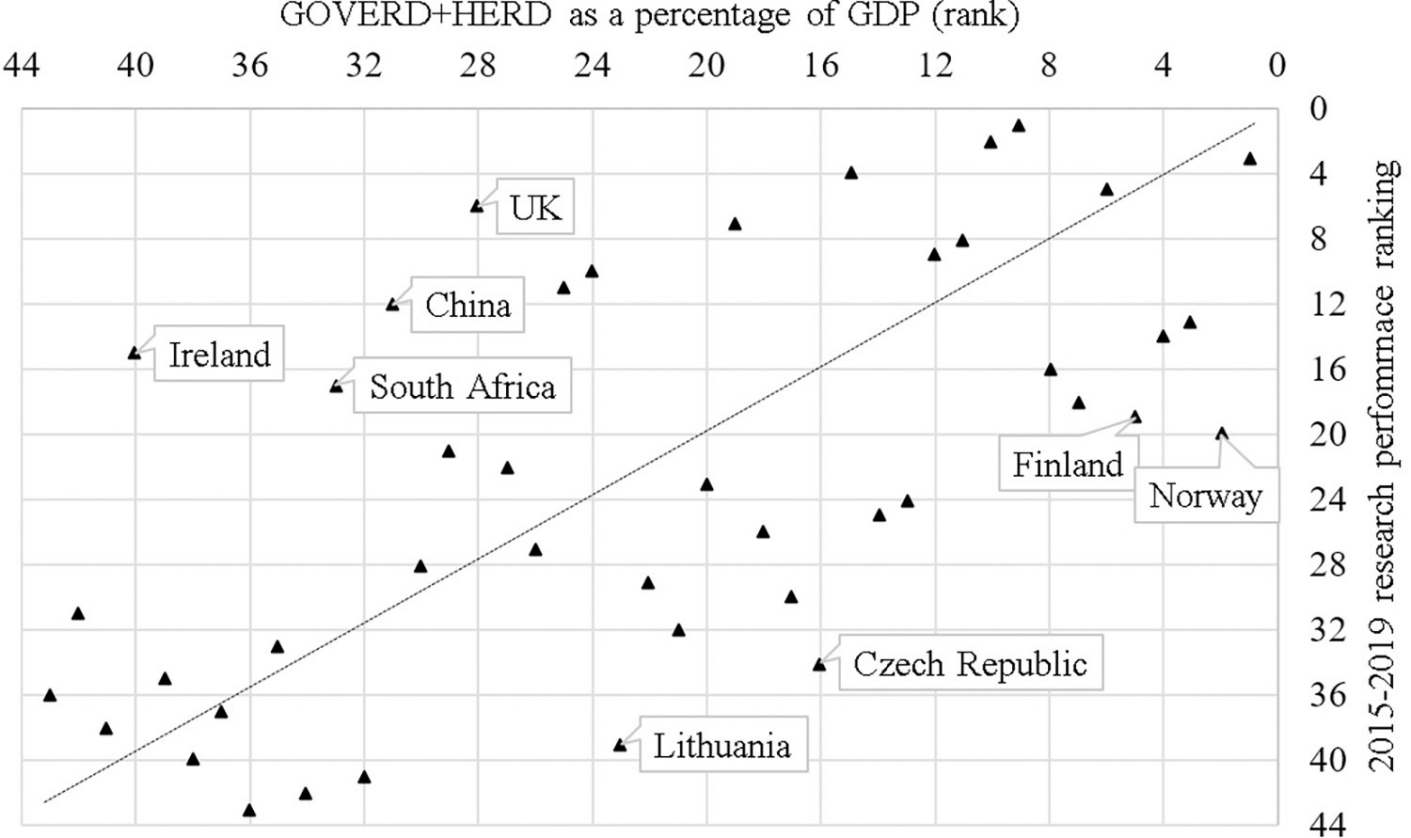
GOVERD+HERD (as a percentage of GDP) and research performance rankings across 43 countries in the dataset (India, Brazil and Costa Rica are not listed in OECD report).

Fig 2 shows the position of each country in terms of scientific standing and propensity to invest in research. A significant correlation occurs between a country’s propensity to invest its wealth in R&D (measured through GOVERD+HERD as a percentage of GDP) and its scientific standing (Spearman rho: 0.610). In particular, among the countries that show a much better ranking in terms of research performance than in terms of propensity to invest in R&D are Ireland, the United Kingdom, China, and South Africa. Conversely, among those that show a better ranking in the propensity to invest in R&D than in research performance, Norway, the Czech Republic, Lithuania, and Finland stand out.

There are several reasons why the scientific standing of a nation is closely intertwined with its economic competitiveness. Countries with strong scientific standing tend to have well-educated and skilled labour forces. This human capital is critical for driving economic growth, as it enables businesses to be more productive and competitive. A solid scientific standing is often associated with a culture of innovation and the development of advanced technologies. Innovation, in turn, is a key driver of economic competitiveness. A robust scientific standing can attract foreign direct investment and business investments. Companies are often attracted to regions with a strong research and innovation ecosystem because they offers access to talent, resources, and a supportive environment for research and development activities. Scientific research contributes to the development of high-quality products and services. When a nation’s scientific community collaborates with industries, it creates goods and services that are more competitive in terms of quality, cost-effectiveness, and innovation. Finally, strong scientific capabilities often lead to economic diversification. A diversified economy is more resilient and competitive because it is not overly reliant on one specific industry or sector. A nation’s scientific capacity can facilitate this diversification by fostering innovation across multiple domains.

We therefore correlated the revealed scientific standing of nations with their economic competitiveness, as measured by the labour productivity as defined and calculated by the International Labour Organization [19]: The average 2015–2019 output per worker (GDP constant 2017 international $ at PPP).

**Fig 3:**
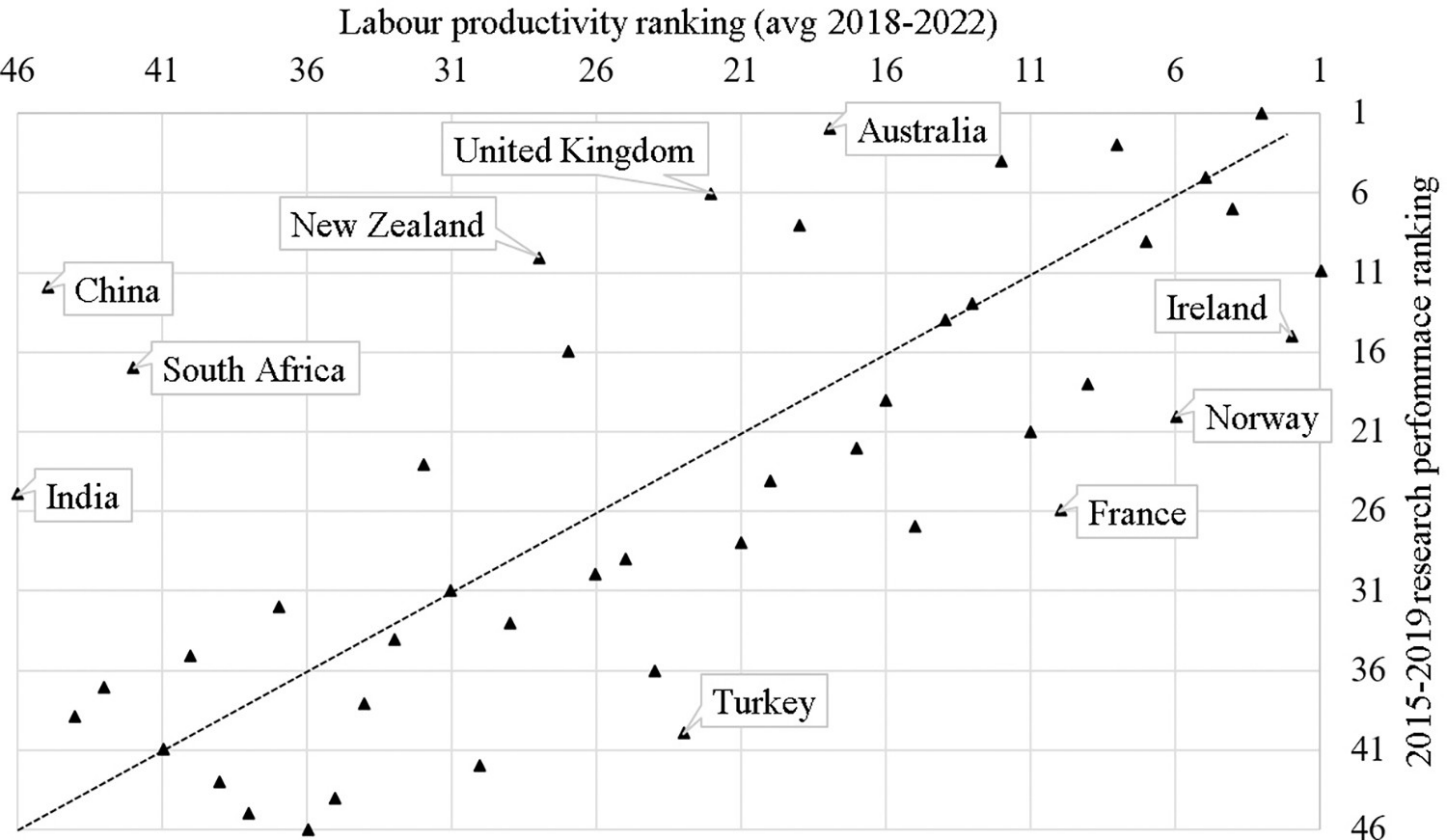
Economic competitiveness (labour productivity) and research performance rankings across countries in the dataset.

The comparison for 46 countries in the dataset, shown in Fig 3, indicates a strong positive correlation (Spearman rho of 0.657) between the two rankings.

In particular, the figure makes it easy to discriminate between countries above and below the bisector. Among those with ranking differences, in absolute value, over twelve positions, we find:

- China, South Africa, India, New Zealand, the UK, and Australia—countries whose ranking, in terms of research performance, is better than that for competitiveness;
- Turkey, France, Norway, and Ireland—countries whose ranking in terms of competitiveness is better than that for research performance.

It should be remembered that translating good scientific performance into equally good economic competitiveness requires ability in cross-sector technology transfer and good absorptive capacity of the production system [45]. In the absence of these requisites, because non-proprietary knowledge is a public good, easily transmitted, and transnational, the country’s scientific production may even benefit competing countries who have better developed these capabilities. Moreover, economic competitiveness can also be supported by factors other than research, such as natural resource endowments, macroeconomic stability, health, infrastructure, ITC adoption, and business dynamism.

This study overcomes the limitations of the methodological approaches and bibliometric indicators previously adopted for measurement of the research competitiveness of nations. Indeed, it is not correct to consider as an indicator of performance i) the sum of publications, because they do not have the same value, i.e. impact on scientific advances; ii) full counting of co-authored publications instead of individual contribution to the publications [46]; iii) the sum of citations, even if field-normalized, because the more recent is the publication date, the weaker is the early citations’ predicting power of overall future citations [44,47,48]; and iv) the mean normalized citations per paper because, among others, a country producing more papers and with all else remaining equal (i.e. inputs, mean citations per paper, etc.), would not improve its performance score [33]. Methodologically, it is also incorrect to measure productive efficiency by dividing research outcome by macro quantities, such as total research staff, R&D expenditures, and the like, because publication intensity and thus total impact vary considerably across research fields [16].

However much the proposed approach overcomes the above limitations, others remain, typical of bibliometric techniques, and must be taken into account when interpreting performance results. Bibliometrics assumes that all new knowledge produced is codified in publications indexed in bibliographic repertories, therefore it excludes publications not indexed in them and tacit knowledge. It also considers citation-based indicators as a measure of scholarly impact, assuming that when a scholar cites a publication they have drawn on it, more or less heavily, for the further advancement of knowledge. But it is known that this is not always true, as unjustified (self) citations may actually occur, as well as uncitedness and undercitation [49,50]. Furthermore, citations are unable to capture impact outside the scientific community.

Finally, the performance indicator adopted is only partially an indicator of productive efficiency, as other input data, such as time spent on research and resources available for researchers to do research, are not available.

Some possible distortions in rankings therefore remain. Indeed, countries with a greater propensity to publish in national language journals not surveyed in Web of Science are disadvantaged. This is the case for Japan [51], China and countries of the former Soviet Union and Eastern bloc [52]. Also disadvantaged are countries with a higher proportion of researchers in the private sector e.g., Japan and the US [53], whose publication intensity is on average lower than that of colleagues in the government and higher education sectors. In fact, in order to maximize the returns on R&D investments, the private sector tends to keep research results proprietary and avoid knowledge spillovers, so as not to favor competitors.

## Supporting information

S1 File Research performance in research fields. (XLSX)

S2 File Research performance in research areas and overall. (XLSX)
